# Time to peak and full width at half maximum in MR perfusion: valuable indicators for monitoring moyamoya patients after revascularization

**DOI:** 10.1038/s41598-020-80036-3

**Published:** 2021-01-12

**Authors:** Adam Huang, Chung-Wei Lee, Hon-Man Liu

**Affiliations:** 1 Department of Biomedical Sciences and Engineering, National Central University, Taoyuan City, Taiwan; 2 Department of Medical Imaging, National Taiwan University Hospital, Taipei, Taiwan; 3 Department of Medical Imaging, Fu Jen Catholic University Hospital, Fu Jen Catholic University, No.69, Guizi Rd., Taishan Dist., New Taipei City, 24352 Taiwan

**Keywords:** Biomedical engineering, Neurovascular disorders, Stroke, Cerebrovascular disorders

## Abstract

Moyamoya disease (MMD) is a chronic, steno-occlusive cerebrovascular disorder of unknown etiology. Surgical treatment is the only known effective method to restore blood flow to affected areas of the brain. However, there are lack of generally accepted noninvasive tools for therapeutic outcome monitoring. As dynamic susceptibility contrast (DSC) magnetic resonance imaging (MRI) is the standard MR perfusion imaging technique in the clinical setting, we investigated a dataset of nineteen pediatric MMD patients with one preoperational and multiple periodic DSC MRI examinations for four to thirty-eight months after indirect revascularization. A rigid gamma variate model was used to derive two nondeconvolution-based perfusion parameters: time to peak (TTP) and full width at half maximum (FWHM) for monitoring transitional bolus delay and dispersion changes respectively. TTP and FWHM values were normalized to the cerebellum. Here, we report that 74% (14/19) of patients improve in both TTP and FWHM measurements, and whereof 57% (8/14) improve more noticeably on FWHM. TTP is in good agreement with Tmax in estimating bolus delay. Our study data also suggest bolus dispersion estimated by FWHM is an additional, informative indicator in pediatric MMD monitoring.

## Introduction

Moyamoya disease (MMD) is a chronic, steno-occlusive cerebrovascular disorder of unknown etiology involving arteries at the base of the brain. It primarily affects children but can also occur in adults. Patients usually present with transient ischemic attack (TIA) or hemorrhagic stroke. The disease was first described in Japan^1^ and is found in other ethnic groups^2^. Treatment of MMD often depends on the aggressiveness of its course but most cases (77%) are treated surgically with the goal to restore blood flow to affected areas of the brain^3^. However, there has been an ongoing controversy regarding the comparative effectiveness analysis of direct, indirect, and combined revascularization procedures^4^. Similarly, generally accepted noninvasive tools for therapeutic outcome monitoring have not been well established in the literature.

Positron emission tomography (PET) with [^15^O]-water or gas radiotracers remains the reference standard for quantifying cerebral blood flow (CBF) in MMD^5–7^. However, radiation exposure makes it difficult to apply repeatedly for pediatric patients. Alternatively, using xenon-enhanced CT to define an index of hemodynamic stress distribution, the ratio of CBF in the lentiform nucleus and cortical MCA territories, was proposed^8–10^. More recently, arterial spin labeling, using magnetically labeled blood as an endogenous tracer, is in scientific investigations and clinical trials to quantify CBF completely noninvasively^6,11–13^. However, it requires further refinement and validation to be considered for applications in clinical practice. Dynamic susceptibility contrast (DSC) magnetic resonance imaging (MRI), the most commonly used MR perfusion imaging technique in the clinical setting, is also proposed to derive nondeconvolution-based time to peak (TTP) parameter for quantifying perfusion delay time changes^14,15^. In addition to the aforementioned approaches, statistical methods such as Bayesian estimation^16^ are also applied to improve CBF assessments in patients with MMD^17^.

Recently, DEFUSE-3^18^ and DAWN^19^ trials have suggested that deconvolution-based time to maximum (Tmax) parameter may be a more effective biomarker for identifying salvageable ischemic tissue from infarct core for selecting acute ischemic stroke patients. Using deconvolution with an arterial input function (AIF) selected from the contralateral middle cerebral artery, Tmax is regarded as an AIF-normalized bolus arrival delay time with an optimal threshold of 6 s for identifying critically hypoperfused tissue across studies and patients^20^. It is natural that Tmax has been examined in several MMD studies^6,21,22^. However, to the best of our knowledge, there has been no prior study of pediatric MMD longitudinal changes using Tmax. One possible reason is that the deconvolution process is sensitive to AIF selection^23^. It is highly challenging for clinicians to select the optimal AIF at any DSC MRI scans consistently given the hemodynamic complexities in MMD^24^.

In this study, we analyzed the hemodynamic transition in cerebral cortex tissue of 19 pediatric patients who were treated with indirect vascularization procedures and had one preoperational and multiple periodic DSC MRI examinations after operation over a time span of 4 to 38 months. Cortical areas of blood perfusion improvement by collateral development (arteriogenesis)^25^ were evaluated by DSC MRI bolus delay and dispersion at a variety of time thresholds. Shortened delay indicates that new faster blood supplying routes are established while narrowed dispersion indicates that more direct routes, possibly via larger arteries, are supplying blood. We took a rigid gamma variate model (GVM) fitting approach^26^ to derive two nondeconvolution-based perfusion parameters: time to peak (TTP) and full width at half maximum (FWHM)^27^ for estimating bolus delay and dispersion respectively. In addition, GVM-derived bolus characteristics also allowed fully automated AIF selection for estimating Tmax^26^. Here we present the bolus delay and dispersion changes in pediatric MMD in terms of TTP, FWHM, and Tmax quantitatively and the correlation agreement analyses between TTP, Tmax, and FWHM by intraclass correlation coefficient (ICC)^28^.

## Results

### DSC-MRI data

The study data consisted of 19 patients (12 boys, 7 girls, no older than 19 years, mean age 10.7 ± 4.6), each with 3 to 8 scans (mean, 6.3 ± 1.4) for periods of time ranging from 4 to 38 months (mean, 16.3 ± 9.2). By excluding 11 low quality scans from statistical analysis, there were in total 108 scans (mean per person, 5.7 ± 1.5). Each patient had at least one of three indirect revascularization procedures: left encephaloduroarteriosynangiosis (EDAS), right EDAS, and multiple burr hole operations. All patients with Suzuki stages range from I to IV^29^. There were 14 ischemic, 5 no-stroke, and 0 hemorrhage cases in MRI imaging findings. Table 1 lists patient information with the chronological sequence of both the DSC-MRI scans and surgical operations. Note that most patients received 2 surgery treatments in different time order. The shortest latest effective scan was 3 months.

**Table 1:** Patient demographics.

Patients	Age	Sex	TIA^†^	MRI^‡^	Left EDAS^#^ (SS)^&^	Right EDAS^#^ (SS)^&^	Multiple Burr hole^#^	DSC-MRI (multiple scans)^#^
1	18	F	No	n	+ 0 (II)	+ 2 (II)		− 5/ + 1/ + 2*/ + 4/ + 5/ + 9
2	19	F	No	i	+ 3 (II)	+ 0 (II)	+ 0	+ 0*/ + 1/ + 3*/ + 5/ + 7/ + 11/ + 14
3	13	F	Yes	i	+ 3 (II)	+ 0 (II)	+ 5	+ 0*^§^/ + 3*/ + 5*/ + 7/ + 12/ + 17/ + 29
4	10	M	Yes	i	+ 0 (III)	+ 6 (II)		+ 0*/ + 1/ + 6*/ + 7^§^/ + 9/ + 13/ + 25
5	9	M	No	i	+ 0 (III)	+ 33 (III)	+ 4	+ 0*^§^/ + 13/ + 16/ + 28/ + 31/ + 34/ + 38
6	13	M	Yes	n	+ 2 (III)	+ 0 (III)		− 1/ + 2*/ + 3/ + 5/ + 8/ + 14/ + 26
7	5	M	Yes	n	+ 0 (II)	+ 1 (II)	+ 4	+ 0*^§^/ + 1*^§^/ + 3/ + 5/ + 7/ + 10/ + 18/ + 23
8	4	F	Yes	i	+ 0 (III)	+ 0.5 (III)	+ 6	+ 0*/ + 2/ + 6*/ + 7/ + 10/ + 15/ + 24
9	13	M	No	n	+ 0 (III)	+ 6 (II)		+ 0*/ + 1/ + 3/ + 6*/ + 7/ + 12/ + 18^§^
10	7	F	Yes	i	+ 0 (II)	+ 6 (II)		+ 0*/ + 1/ + 3/ + 6*/ + 7/ + 9/ + 12/ + 18
11	5	M	Yes	i	+ 0 (III)	+ 2 (III)		+ 0*^§^/ + 2*/ + 3/ + 5^§^/ + 8/ + 14
12	11	M	Yes	i	+ 0 (II)	+ 5 (I)		+ 0*/ + 1/ + 3/ + 5*/ + 6/ + 8/ + 11/ + 17
13	6	M	Yes	i	+ 0 (II)	+ 9 (I)		+ 0*/ + 1/ + 4/ + 8/ + 10/ + 12
14	13	F	Yes	n	(II)	+ 0 (II)		+ 0*/ + 1/ + 3^§^/ + 7
15	5	M	Yes	i	+ 0 (IV)	+ 3 (III)		+ 0*/ + 1/ + 3*/ + 5/ + 7/ + 9
16	13	M	Yes	i	+ 0 (II)	(I)	+ 3	+ 0*/ + 1/ + 3*/ + 4/ + 6^§^/ + 9
17	12	M	No	i	+ 5 (I)	+ 0 (II)		+ 0*/ + 1/ + 3/ + 6/ + 8
18	10	M	Yes	i	+ 6 (II)	+ 0 (II)		+ 0*/ + 1/ + 3/ + 6*^§^
19	18	F	Yes	i	(IV)	+ 0 (IV)		+ 0*/ + 1/ + 4

^†^Patient presents with transient ischemic attack (TIA).

^‡^MRI imaging findings: no stroke (n), ischemic (i), or hemorrhage (h).

^&^Suzuki stage (SS).

^#^The action time in *i* months after the first surgical operation (assigned with a ‘ + *i*’ timestamp) of left encephalo-duro-arterio-synangiosis (EDAS), right EDAS, or burr hole operation.

*DSC-MRI scanning was performed a few days before a surgical operation.

^§^Eleven low quality scans that were not included in statistical analysis.

### Nondeconvolutional perfusion parameter maps

**Figure 1:**
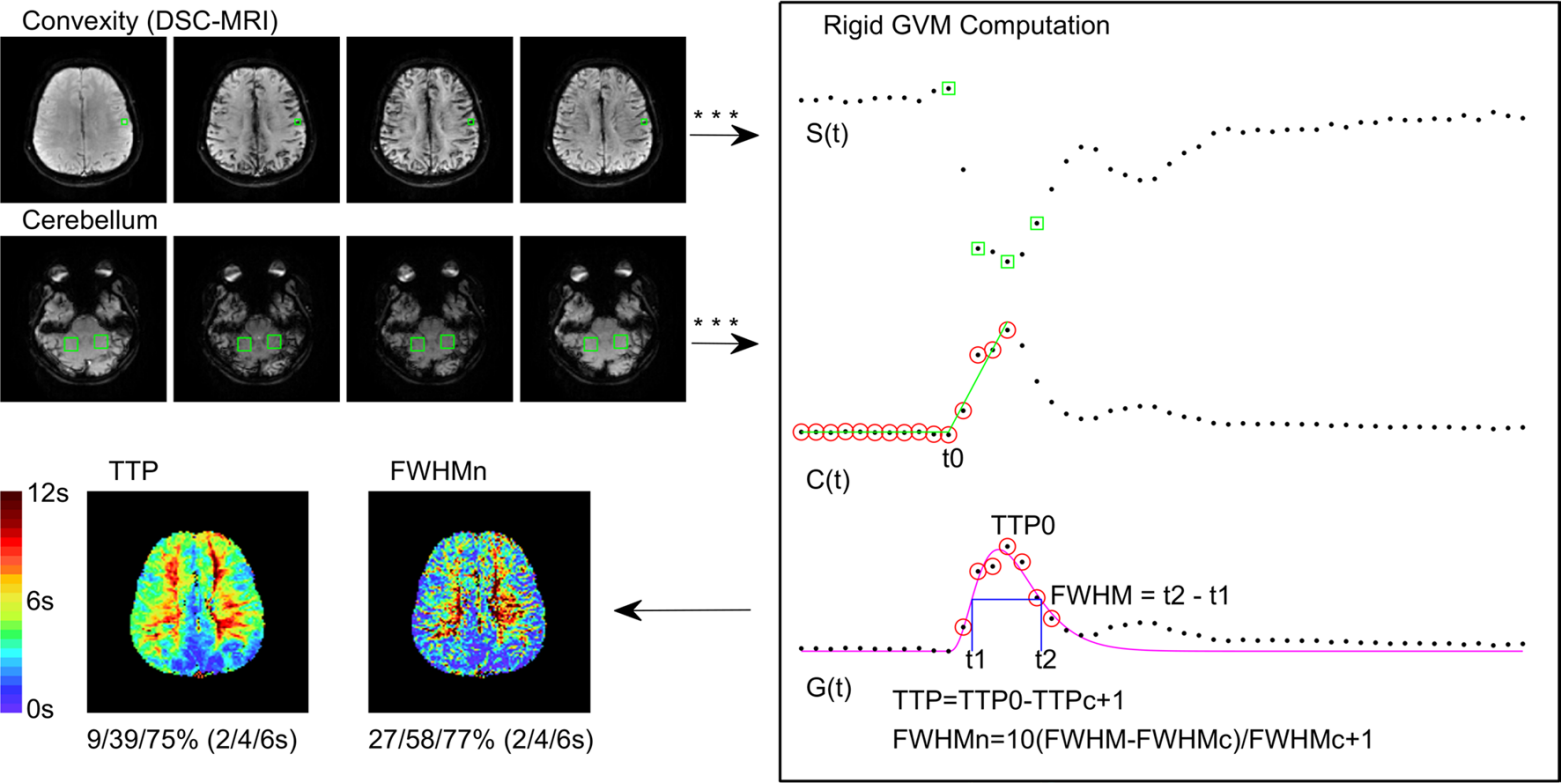
Derivation of TTP and FWHM perfusion maps (patient 1, the earliest scan). Brain tissues in the convexity slice (S(t) is sampled at the small box) and manually selected cerebellum references (green boxes) are computed by the proposed rigid GVM approach. In rigid GVM computation, green squares indicate the sampling time of four MRI images on the left and red circles indicate data points used in model fitting. TTP_0_ and FWHM are normalized using the listed empirical formulas and color-coded from 0 to 12 s. Normalized areas (in percentage) at thresholds of 2, 4, and 6 s are given under each map.

Nondeconvolution-based MR perfusion parameters TTP and FWHM were estimated for all 119 DSC MRI scans (including low quality scans) listed in Table 1. For computational simplicity and consistency, the estimations were done only on an image slice near convexity as removal of cerebrospinal fluid was not required in this region. Figure 1 illustrates the image processing procedure and the resultant TTP and FWHM maps.

The computation in Fig. 1 is summarized as follows. Assuming a linear relationship between the tissue contrast agent concentration and the change in T2* relaxation rate, contrast concentration time curves *C*(*t*) were computed on a voxel-by-voxel basis without smoothing by:

1 $$ C\left( t \right) = - {\text{log}}\left( {\frac{S\left( t \right)}{{S_{0} }}} \right)/TE $$

where *S*(*t*) denoted MR signal intensity time curves, *S*_0_ the precontrast signal intensity, and *TE* the echo time. Let the maximal *S*(*t*) be *S*_0_ and *TE* = 1 for computational simplicity. Level *C*_0_ and bolus arrival time *t*_0_ of *C*(*t*) were decided by a linear–linear model^30^. The level-adjusted concentration *G*(*t*) was modeled by:

2 $$ G\left( t \right) = C\left( {\text{t}} \right) - C_{0} = A\left( {t - t_{0} } \right)^{\alpha } \exp \left( { - \frac{{{\text{t}} - t_{0} }}{{\upbeta }}} \right), $$

a GVM with four parameters *A*, *t*_0_, α, and β, where (TTP – *t*_0_) = αβ by definition. The number of free parameters in *G*(*t*) were reduced to 2 by fixing the values of *t*_0_ and TTP^31^. *G*(*t*) was solved efficiently by linear regression using data points between *t*_0_ to *t*_2_ (*G*(*t*_2_) = half maximum)^26,31^.

TTP and FWHM were derived in high precision using the fitted model *G*(*t*) defined by Eq. (2) (see Supplementary Information for estimation error analysis). Note that in order to compare inter-scan results, all the TTP and FWHM were normalized by referring to the mean TTP_c_ and FWHM_c_ (subscription c for cerebellum) of the green boxes at cerebellum manually selected by operators. TTP and FWHM normalization formulas were empirically selected such that TTP, FWHM, and Tmax perfusion color maps were in good agreement. (Refer to Methods Section for further details).

**Figure 2:**
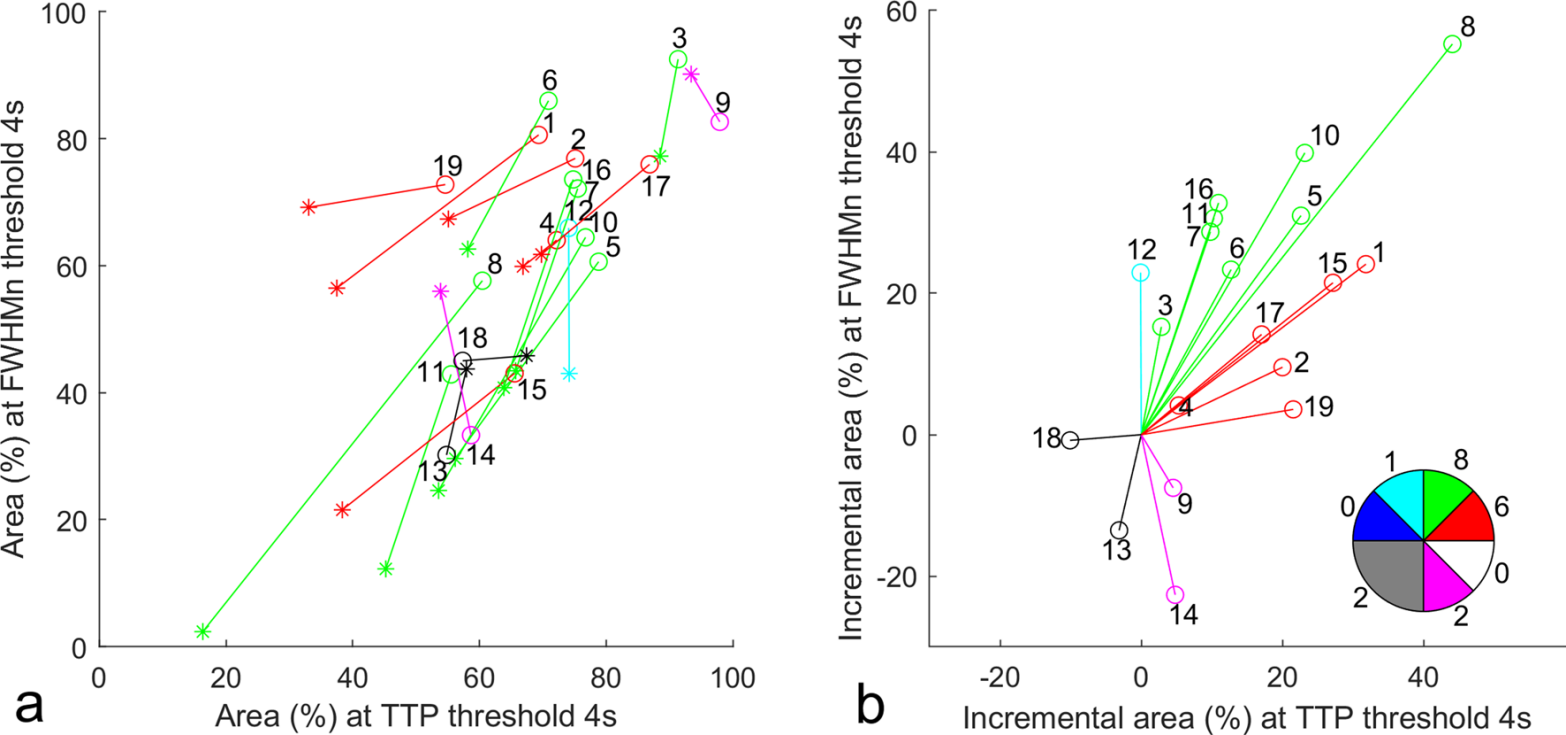
(**a**) TTP and FWHM_n_ of 19 patients of the earliest (star) and latest clean scans (circle) at threshold under 4 s (in percentage of the convexity area). (**b**) The earliest points (star) are rearranged to locate at the origin to show area increments (latest–earliest). All the vectors in (**a**) and (**b**) are color-coded according to the vector angle illustrated in the pie chart in (b). Patient number is marked next to the circle of each vector, and the population of vectors in each color is given around the pie chart.

Figure 2 illustrates the resultant TTP and FWHM_n_ areas estimated at 4-s threshold of the earliest and latest clean DSC MRI scans of all patients. Figure 2b shows, in a cohort of 19 pediatric MMD patients treated with indirect revascularization procedures, 17 (89%) patients show shorter TTP (area under a 4-s threshold increased), 15 (79%) narrower FWHM_n_, and 14 (74%) both. Among the 14 patients with shorter TTP and narrower FWHM_n_, 57% (8/14) improve more noticeably on FWHM_n_.

### Longitudinal monitoring examples

**Figure 3:**
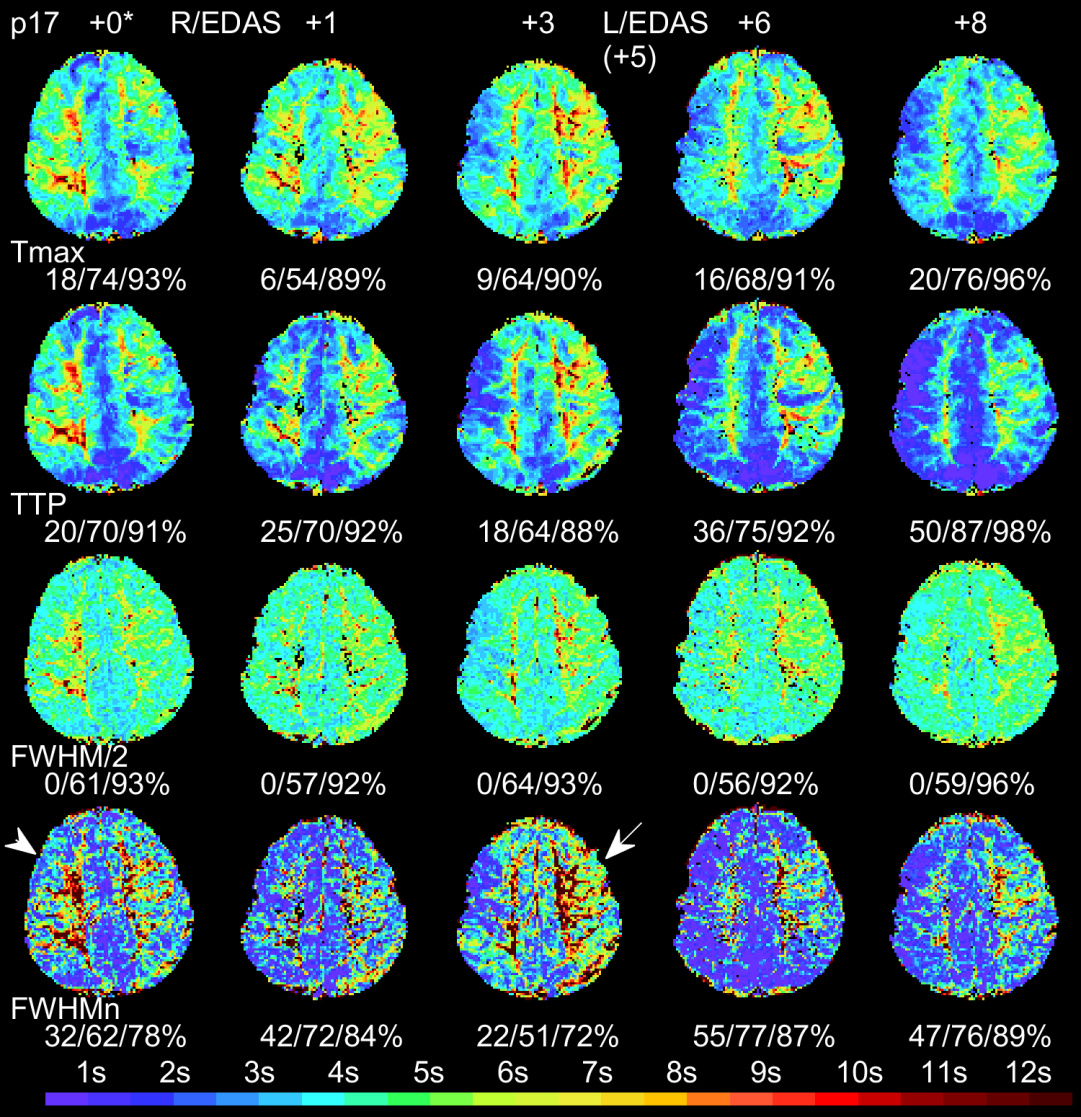
Representative perfusion maps of a 12-year-old boy (patient 17 in Table 1) who underwent a right EDAS operation first and a left EDAS five months later. Normalized delay time areas at thresholds of 2, 4, and 6 s are given under each map. Tmax (first row, derived by SVD_+M_) shows modest perfusion improvement while TTP (second row) indicates more improvement. FWHM/2 (third row) shows consistent pattern in green color. FWHM_n_ (fourth row) becomes narrower gradually on the right hemisphere (arrow head) but wider at month + 3 on the left hemisphere (arrow), and narrower thereafter.

Figure 3 shows an example of a 12-year-old boy (patient 17 in Table 1 and Fig. 2) who underwent right EDAS procedure first and left EDAS five months later. While the FWHM/2 (rescaled, half-FWHM) by sequence appears consistent, the normalized FWHM_n_ clearly indicates that tissue bolus time curves on the right hemisphere become narrower gradually but those on the left become wider at month + 3, and narrower thereafter. Tmax and TTP also show similar longitudinal patterns.

**Figure 4:**
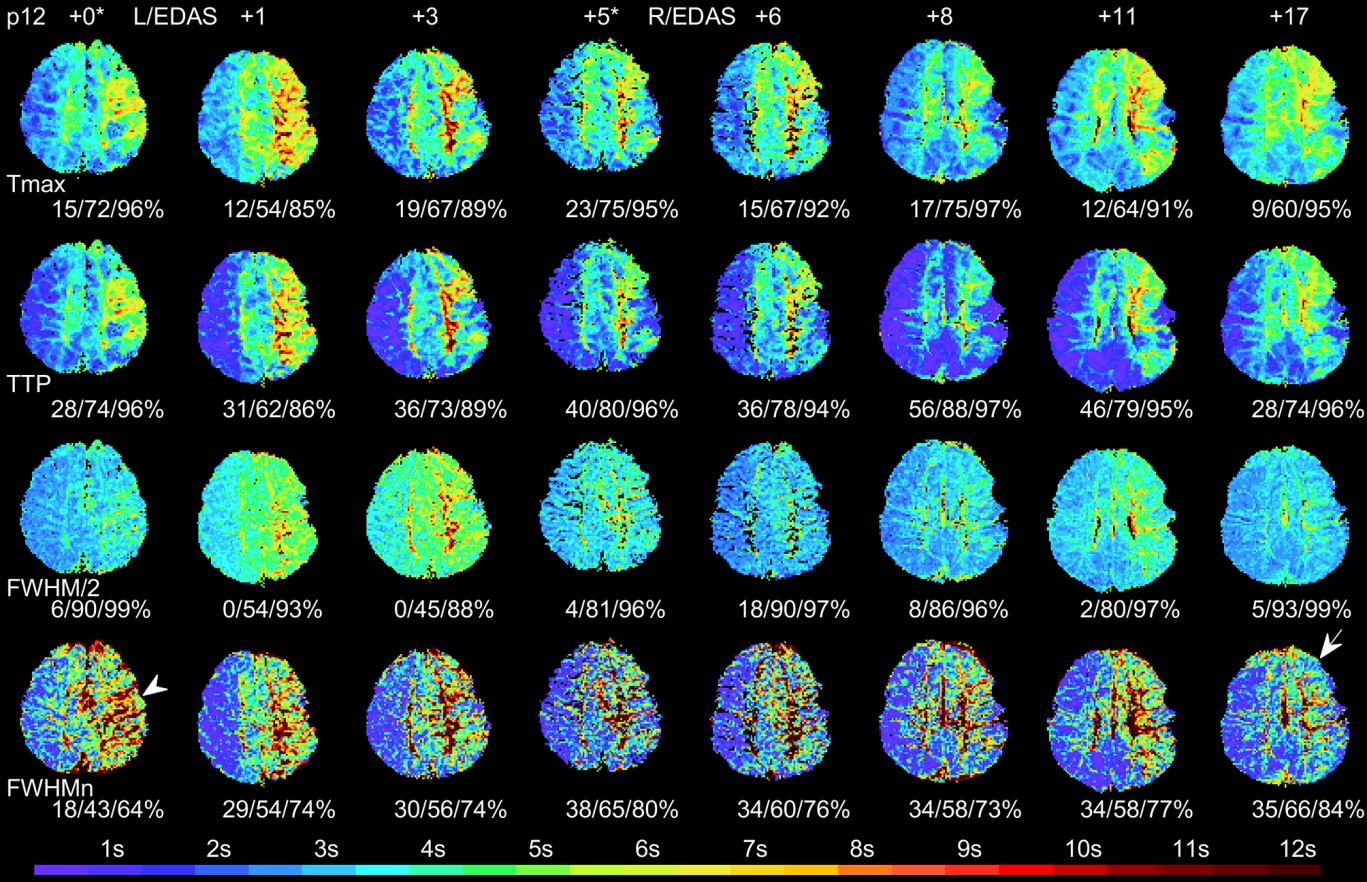
Representative perfusion maps of an 11-year-old boy (patient 12 in Table 1) who underwent a left EDAS operation first and a right EDAS five months later. Normalized delay time areas at thresholds of 2, 4, and 6 s are given under each map. Both Tmax (derived by SVD_+M_) and TTP show that perfusion on the left hemisphere improved between months + 5* to + 8 but returned to the pre-surgery condition (+ 0*) after month + 11. However, FWHM_n_ indicates that the contrast time curves on the left hemisphere have become narrower (arrow) compared to pre-surgery (arrow head).

**Figure 5:**
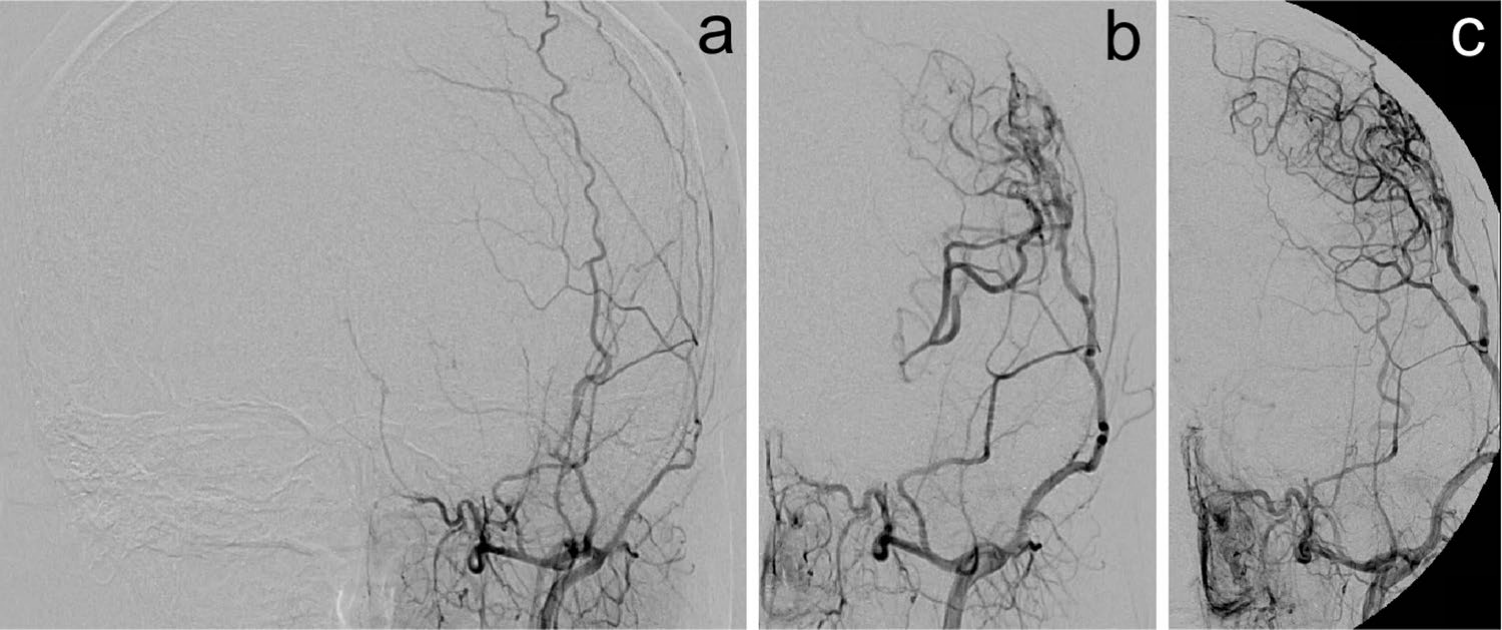
Digital subtraction angiography (DSA) images of the 11-year-old boy with MR perfusion maps shown in Fig. 4. (**a**) Anterior projection from external carotid artery injection right before the left EDAS operation in month + 0. (**b**) DSA performed in month + 5 shows moderate collateral development. (**c**) DSA image performed in month + 17 shows significant collateral development compared to (**b**) in the upper area.

Figure 4 is another example of an 11-year-old boy (patient 12 in Table 1 and Fig. 2) who underwent left EDAS procedure first and right EDAS five months later. TTP and Tmax maps at + 0 (a few days before the first operation) and + 17 months appear similar as if the delay time parameters only fluctuate without major changes. However, FWHM_n_ maps illustrate that the left hemisphere progresses consistently throughout the observation time window with observable lowering FWHM_n_ even 17 months after the initial left EDAS operation. Digital subtraction angiography (DSA) images of the same patient performed in months + 0 (before left EDAS procedure), + 5 (after right EDAS procedure), and + 17 (follow-up) are illustrated in Fig. 5. The DSA patterns suggest that the FWHM_n_ parameter has an inverse relationship with arteriogenesis stimulated by the left EDAS procedure.

### Correlation agreement between TTP and Tmax

We conducted a set of eight correlation agreement analyses between the proposed rigid GVM-derived TTP and eight different Tmax derivations using all 108 clean DSC MRI scans. The full ICC results of the TTP and Tmax areas at time thresholds 2, 3, 4, 5, and 6 s at convexity are listed in Table 2 respectively.

**Table 2:** ICC agreement analysis between Tmax/FWHM and TTP using the normalized delay time areas at thresholds of 2, 3, 4, 5, and 6 s.

MRP*	≤ 2 s ICC (95% CI)	≤ 3 s	≤ 4 s	≤ 5 s	≤ 6 s
FFT_−A_	0.27 (0, 0.48)	0.48 (0.04, 0.71)	0.62 (0.13, 0.81)	0.71 (0.29, 0.86)	0.77 (0.42, 0.89)
FFT_−M_	0.34 (0.09, 0.53)	0.56 (0.28, 0.73)	0.69 (0.45, 0.82)	0.78 (0.59, 0.87)	0.82 (0.69, 0.89)
FFT_+A_	0.60 (0.46, 0.71)	0.62 (0.49, 0.72)	0.73 (0.60, 0.82)	0.77 (0.62, 0.86)	0.86 (0.77, 0.91)
FFT_+M_	0.60 (0.46, 0.70)	0.60 (0.43, 0.73)	0.70 (0.42, 0.83)	0.75 (0.49, 0.87)	0.85 (0.69, 0.92)
SVD_−A_	0.36 (0, 0.61)	0.43 (0, 0.70)	0.56 (0.03, 0.79)	0.70 (0.19, 0.86)	0.78 (0.43, 0.90)
SVD_-M_	0.45 (0.13, 0.66)	0.55 (0.09, 0.76)	0.66 (0.28, 0.82)	0.79 (0.55, 0.89)	0.84 (0.70, 0.90)
SVD_+A_	0.42 (0.11, 0.63)	0.63 (0.35, 0.78)	0.75 (0.64, 0.83)	0.85 (0.78, 0.89)	0.90 (0.85, 0.93)
SVD_+M_	0.50 (0.28, 0.66)	0.67 (0.53, 0.77)	0.79 (0.70, 0.85)	0.85 (0.78, 0.89)	0.90 (0.84, 0.93)
FWHM/2	0 (0, 0.08)	0.09 (0, 0.24)	0.28 (0.10, 0.44)	0.43 (0.26, 0.57)	0.57 (0.43, 0.69)
FWHM_n_	0.63 (0.39, 0.77)	0.69 (0.58, 0.78)	0.61 (0.32, 0.77)	0.50 (0.01, 0.75)	0.43 (0, 0.71)

* MRP stands for the estimated MR perfusion parameters Tmax and FWHM. Tmax estimated by different deconvolution methods are noted with FFT, SVD with ( +) and without (−) GVM preprocessing; the characters ‘A’ and ‘M’ stand for the average and median of Tmax estimates derived from the top three AIFs respectively.

The deconvolution for estimating Tmax was implemented by using Singular Value Decomposition (SVD) in time domain^32^ and Fast Fourier Transform (FFT) in frequency domain^33^ and noted as FFT-, FFT + , SVD-, and SVD + , where ‘ + ’ and ‘−’ representing with ( +) and without (−) using the proposed GVM preprocessing. Three top-ranked AIFs were automatically selected by in-house software^26^ to derive three Tmax estimates for each FFT and SVD derivation. These three estimates were then used to derive the average (A) and median (M) Tmax at each voxel. In total, Table 2 lists eight Tmax’s which are noted as FFT_-A_, FFT_-M_, FFT_+A_, FFT_+M_, SVD_-A_, SVD_-M_, SVD_+A_, and SVD_+M_ according to their derivation approaches.

**Figure 6:**
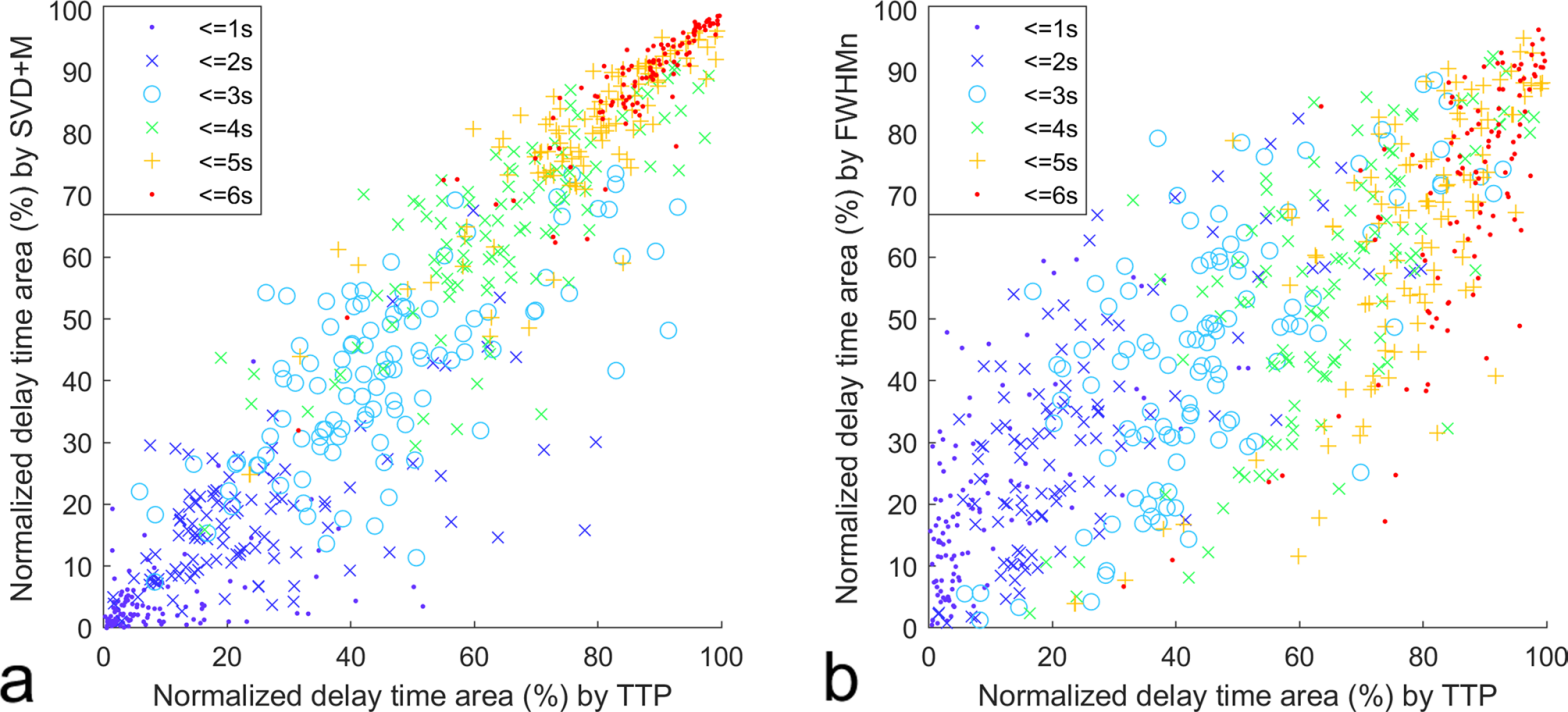
Scatterplots of normalized delay time areas at thresholds of 1, 2, 3, 4, 5, and 6 s (color-coded accordingly) with TTP as × coordinate and (**a**) Tmax estimated by SVD_+M_, (**b**) FWHM_n_ as y coordinate.

The level of agreement was interpreted by the guidelines proposed by Koo et al.^28^: ICC < 0.50 (poor), 0.5–0.75 (moderate), 0.75–0.90 (good), and > 0.90 (excellent). Poor to moderate agreement was found between all eight Tmax’s and TTP at thresholds 2 and 3 s for most computation settings in Table 2. Only one case had moderate to good agreement with TTP at threshold 3 s. It was SVD_+M_ with ICC 0.67 (95% confidence intervals (CI): 0.53, 0.77). Overall, Tmax estimated by SVD_+A_ and SVD_+M_ were in best agreement with TTP. SVD_+M_ was above the average at thresholds 4 (ICC 0.79; 95% CI: 0.70, 0.85), 5 (ICC 0.85; 95% CI: 0.78, 0.89), and 6 (ICC 0.90; 95% CI: 0.84, 0.93) seconds, which were moderate to good, good, and good to excellent respectively. Figure 6a shows the correlation scatterplots for TTP and Tmax (derived by SVD_+M_) at thresholds 1, 2, 3, 4, 5, and 6 s.

### Correlation agreement between TTP and FWHM

Figure 6b shows the correlation scatterplots for TTP and FWHM_n_ at thresholds 1, 2, 3, 4, 5, and 6 s respectively. The data points are more scattered in Fig. 6b as compared with Fig. 6a. The ICC agreement evaluations between perfusion delay time areas estimated by FWHM/2, FWHM_n_, and TTP at thresholds 2, 3, 4, 5, and 6 s are listed in the end of Table 2, where FWHM is divided by 2 (noted FWHM/2) for scaling the time measurements down to a range roughly between 0 and 6 s. The highest ICC was 0.57 (95% CI: 0.43, 0.69) at time threshold 6 s for FWHM/2 and 0.69 (95% CI: 0.58, 0.78) at time threshold 3 s for FWHM_n_ respectively. FWHM_n_ is in poor to moderate agreement, but FWHM/2 is prominently in poor agreement with TTP.

## Discussion

TTP and Tmax are two popular perfusion parameters in monitoring cerebral perfusion changes after revascularization in patients with MMD using DSC MRI. Both parameters are shown to be sensitive enough to monitor transitional bolus delay time changes in several studies^6,14,15,21,22^. Recent large-scale multi-center trials have suggested that the deconvolution-based Tmax parameter may be a more effective biomarker with an optimal threshold of 6 s for identifying critically hypoperfused tissue across studies and patients^18–20^. However, deconvolution-based Tmax is known to be sensitive to AIF selection. It is particularly challenging for longitudinal MMD monitoring as the contralateral side is rarely clear given a MMD patient with multiple operations at different time.

To alleviate the uncertainty of AIF selection and to serve as an alternative viewpoint, we proposed using a rigid GVM approach to derived cerebellum-normalized bolus delay (TTP) and dispersion (FWHM_n_) parameters for longitudinal MMD monitoring, assuming no vertebral arterial abnormality existed to affect cerebellum circulation. The resultant TTP was statistically found to be in good agreement with Tmax in delay time map area measurement (Figs. 3, 4, 6a). The resultant FWHM_n_ was shown to be in less agreement with TTP (Figs. 3, 4, 6b) but equally sensitive to detect bolus contrast time curve improvement. In Fig. 2b, there are 17 (89%) patients showing shorter TTP and 15 (79%) narrower FWHM_n_, and 14 (74%) both. Among the 14 patients with shorter TTP and narrower FWHM_n_, 57% (8/14) improve more noticeably on FWHM_n_. Patients 15 and 19 with Suzuki grades of IV have shown noticeable improvement in Fig. 2. Therefore, our method is likely available for Suzuki grades between I and IV. Regarding patients 13 and 18, who had no improvement in either TTP or FWHM_n_, we notice that both patients had operation performed within 3 months right before the latest DSC-MRI scans. We need further scans to evaluate these two cases more accurately. (See Supplementary Information for the perfusion maps of patients 13 and 18).

Note that a variety of Tmax implementations exists and FWHM_n_ does not always change in accordance with TTP (Table 2), perfusion delay time parameters for MMD monitoring should be implemented and interpreted with a few caveats discussed in the following.

First, using a rigid GVM preprocessing approach to reduce scan noise-caused error, the fitted model in Eq. (2) is more robust and precise for deriving both bolus delay (TTP) and dispersion (FWHM) estimations. In addition, GVM can remove the recirculation effect from DSC MRI data for deconvolution computation. As a result, Table 2 shows that Tmax estimates by FFT_+_ and SVD_+_ are in better agreement with TTP than FFT_-_ and SVD_-_ respectively. Nonetheless, we recommend applying SVD_+_ only for numerical stability reasons because the resultant *G*(*t*) defined by Eq. (2) could cause wiggles in some FFT_+_ computations.

Second, using an empirical formula: TTP = TTP_0_—TTP_c_ + 1, we were able to normalize raw TTP_0_ to the similar time range of Tmax with an optimal threshold of 6 s by referring to cerebellar TTP_c_ estimated from manually selected cerebellar patches. The “ + 1” adjustment was empirically decided to achieve best ICC scores for all tested Tmax computations. Tmax estimated by SVD_+M_ was in moderate to excellent agreement with TTP at delay time thresholds between 4 and 6 s that was of clinical importance. The normalization formula of FWHM_n_ was empirically decided in a similar fashion.

Third, the clinical indications and interpretations of Tmax or TTP should not be conducted alone without comparing with other perfusion parameters. As a reminder, the scans (Fig. 4) of patient 12 at scanning time + 0 and + 17 months may appear similar to human eyes. Without reviewing other perfusion parameters, the radiological diagnosis might state that the TTP and Tmax delay time maps were fluctuating but stable without major changes. However, FWHM_n_ illustrated a complete different scenario that the left hemisphere progressed consistently throughout the observation time window with observable FWHM_n_ narrowing. By reviewing DSA images (Fig. 5), we found that FWHM_n_ narrowing agreed with local blood flow improvement in an apparently inverse relationship.

Our study had two main limitations. First, we did not validate the accuracy of the automatically detected AIFs because accurate determinations of true AIF were challenging for MMD patients who were in different disease stages and underwent a variety of revascularization procedures. Instead, we conducted a series of ICC analyses between TTP and 8 different Tmax derivations with 3 automatically top-ranked AIFs. The statistical ICC analyses indicated that the automatically selected AIFs were relatively consistent and accurate for Tmax estimations. Second, our data size was too small for statistical grouping analysis with age, sex, associated disease stages, operative methods, and clinical results. In spite of the aforementioned limitations, our results indicated that nondeconvolution-based time to peak and peak width changes were observable perfusion characteristics by using cerebellar normalization.

In conclusion, Good to excellent agreement between time-to-maximum and time-to-peak MR perfusion delay time evaluations of pediatric moyamoya disease can be achieved by using a rigid gamma variate model fitting approach. Our study data also suggest bolus dispersion estimated by properly normalized FWHM may be an additional, informative indicator in pediatric MMD monitoring. The innovation of this study can be applied to clinical practice directly without changing current DSC-MRI scanning protocols.

## Methods

This study and all experimental protocols were approved by the institutional review board of National Taiwan University Hospital, Taipei, Taiwan. Written informed consent was obtained from all participants and the children’s parents. All methods were carried out in accordance with relevant guidelines and regulations.

### Materials

We retrospectively reviewed a cohort of 81 MMD patients with DSC MRI scans performed from September 2011 to January 2015. A subgroup of 49 patients aged no older than 19 years were initially selected. Among them, 38 patients had at least one of three indirect revascularization procedures: left EDAS, right EDAS, and multiple burr hole operations. Of these 38 patients, 2 without pre-surgery and 17 without at least two post-surgery scans were excluded.

### Imaging protocol

All patients were imaged on a 1.5-T MR imaging unit (Signa HDx, GE Healthcare, Milwaukee, Wis), using gradient-echo DSC MRI with the following parameters: flip angle, 90 degrees; acquisition matrix, 128 × 128; pixel spacing, 1.875 × 1.875 mm; slice thickness, 5 mm; slice gap, 1 mm; echo time (msec)/repetition time (msec), 40/2000; number of temporal positions, 50; and 11–25 slices (mean, 20.5 ± 2.6) covering at least half of the cerebellum to the top of the cerebrum. Gadovist (gadobutrol; 0.1 mmol/kg; Bayer Schering Pharma, Berlin, Germany) was injected 5 s after the DSC MRI commenced at 3 cm^3^/s injection rate and followed by a 20-cm^3^ saline flush. The raw DSC MRI data was converted to contrast concentration using Eq. (1) and preprocessed by conventional image segmentation procedures to remove non-brain tissues.

### Rigid GVM fitting

It is possible to do least-squares fitting of Eq. (2) directly, however, it requires a multiple linear regression strategy which is computationally more expensive and numerically less stable as exponential functions are involved. To simplify the computation and make the GVM fitting more robust, here we present a 2-stage rigid GVM computation strategy, which is illustrated as C(t) and G(t) in Fig. 1.

#### Stage 1

The precontrast level *C*_0_ and the bolus arrival time *t*_0_ in Eq. (2) were estimated by a linear–linear-model LLM(t). In this model, the portion of contrast concentration time curves *C*(*t*) for $0\le t\le TTP{^{'}}$ (red circled points in *C*(*t*) with the maximum at *t* = *TTP’*, Fig. 1) were approximated by a linear–linear piecewise function^26,30^:

3 $$ LLM\left( t \right) = \left\{ {\begin{array}{*{20}l} {C_{0} } \hfill & {0 \le t \le t_{0} } \hfill \\ {C_{0} + C_{1} \left( {t - t_{0} } \right) } \hfill & {t_{0} < t \le TTP^{'}} \hfill \\ \end{array} } \right. $$

such that the sum of squared error SSE(*t*_0_)

4 $$ SSE\left( {t_{0} } \right) = \mathop \sum \limits_{t = 0}^{TTP^{'}} \left( {LLM\left( t \right) - C\left( t \right)} \right)^{2} $$

was minimized. Substituting *t* by *x* in Eq. (3) where *x* = 0 if $0\le t<{t}_{0}$ and *x* = *t*—*t*_0_ if ${t}_{0}<t\le TTP{^{'}}$ , Eq. (3) can be simplified as :

5 $$ LLM\left( x \right) = C_{0} + C_{1} \times x. $$

Parameters C0 and C1 in Eq. (5) can be solved very efficiently by a linear least-squares estimation. Therefore, Eq. (4) can be minimized by linearly searching along *t*_0_ from 0 to *TTP’* with an appropriate small increment.

#### Stage 2

The level-adjusted concentration *G*(*t*) in Eq. (2) was commonly modeled by four parameters *A*, *t*_0_, α, and β:

$$ G\left( t \right) = C\left( {\text{t}} \right) - C_{0} = A\left( {t - t_{0} } \right)^{\alpha } \exp \left( { - \frac{{{\text{t}} - t_{0} }}{{\upbeta }}} \right), $$

where (TTP − *t*_0_) = αβ by definition. The number of free parameters in *G*(*t*) can be reduced to 2 by fixing the values of *t*_0_ and TTP^26,31^. Rearrange *G*(*t*) with *t*’ = (*t*—*t*_0_)/(TTP − *t*_0_) and *G*_max_ = *A*(TTP—*t*_0_)^α^exp(-α) at *t* = TTP,

6 $$  G(t^{'} ) = G_{{\max }} t^{'} \alpha \exp (\alpha (1 - t')). $$

Take the natural logarithm of both sides of *G*(*t’*):

7 $$ \ln \left( {G\left( {t^{'}} \right)} \right) = \ln \left( {G_{max} } \right) + \alpha \left( {1 + \ln \left( {t^{'}} \right) - t^{'}} \right) $$

The new formula has the form of *y* = *b* + *ax*, where *G*_max_ and α can be determined from linear regression of the natural logarithm of the observed values. In this model, the portion of contrast concentration time curves *G*(*t*) for ${t}_{0}<t\le \sim {t}_{2}$ (red circled points between *t*_0_ and roughly estimated *t*_2_ in *G*(*t*), Fig. 1) were approximated. We name this approach as rigid GVM because *t*_0_ and TTP are fixed in each regression model.

### Perfusion parameter estimation

MR perfusion parameter estimation was done on an image slice near convexity as removal of cerebrospinal fluid was not required in this region. On this convexity image slice, absolute areas were assessed at delay time thresholds 1, 2, 3, 4, 5 and 6 s respectively and normalized by the total cortical area. Tissue bolus-derived delay time parameters Tmax, TTP, and dispersion parameter FWHM are delineated as follows.

Tmax was estimated by Fast Fourier Transform (FFT) in frequency domain^33^ and Singular Value Decomposition (SVD) in time domain^32^, with and without GVM preprocessing. The resultant residue curves were up-sampled at 0.5 s time resolution to find Tmax^33^. The top three AIF candidates detected by a fully automated algorithm^26^ were used to compute three individual Tmax estimates at each pixel. The mean (average) and median values of these three estimates were also computed and analyzed.

TTP was determined as the best fitting result to the rigid GVM, ln(*G*(*t’*)) defined by Eq. (7). For computational speed, we kept the LLM-solved *t*_*0*_ fixed and only conducted linear research for TTP in the neighborhood of the maximal *C*(*t*) with a time step of 0.1 s. The raw result, TTP_0_, was normalized by subtracting the average result, TTP_c_, estimated at manually selected cerebellum areas:

8 $$ {\text{TTP}} = {\text{TTP}}_{0} - {\text{TTP}}_{{\text{c}}} + {1}. $$

The adjustment of 1 s was determined empirically to correlate well with Tmax.

FWHM, peak width measured at half maximum (FWHM = *t*_2_—*t*_1_ as illustrated in Fig. 1), was derived from the resultant GVM. In addition, a normalized version FWHM_n_ referring to FWHM estimated in the cerebellum was also defined empirically as

9 $$ FWHM_{n} = 10\frac{{FWHM - FWHM_{c} }}{{FWHM_{c} }} + 1. $$

All GVM preprocessing and delay time computation were performed by in-house software implemented on the Matlab platform (version 2019b; Mathworks, Natick, MA).

### Statistical analysis

Intraclass correlation coefficient (ICC) (two-way mixed model for absolute agreement, single measurement) was performed to determine agreement between TTP, Tmax, and FWHM. ICC values were interpreted by the proposed guidelines of Koo et al.^28^: < 0.50 (poor), 0.5–0.75 (moderate), 0.75–0.90 (good), and > 0.90 (excellent). All statistical analyses were performed with Matlab (version 2019b; Mathworks, Natick, MA).

## Supplementary Information

Supplementary Information.

## Data Availability

The datasets for this study are protected patient information. Some data may be available for research purposes from the corresponding author upon reasonable request.

## Code availability

Matlab codes and data for generating Fig. 1 are openly available on Github website at: https://github.com/hyadamhuang/DSC-MRI-TTP. It consists of one DSC-MRI dataset (including convexity and cerebellum) and the Matlab codes for deriving GVM, TTP, and FWHM.

## References

[1] Suzuki J, Takaku A (1969). Cerebrovascular “moyamoya” disease. Disease showing abnormal net-like vessels in base of brain. Arch. Neurol..

[2] Starke RM (2012). Moyamoya disorder in the United States. Neurosurgery.

[3] Burke GM (2009). Moyamoya disease: a summary. Neurosurg. Focus..

[4] Teo MK, Madhugiri VS, Steinberg GK (2017). Editorial: direct versus indirect bypass for moyamoya disease: ongoing controversy. J. Neurosurg..

[5] Vakil P, Lee JJ, Mouannes-Srour JJ, Derdeyn CP, Carroll TJ (2013). Cerebrovascular occlusive disease: quantitative cerebral blood flow using dynamic susceptibility contrast MR imaging correlates with quantitative H2[15O] PET. Radiology.

[6] Hara S (2017). Noninvasive evaluation of CBF and perfusion delay of moyamoya disease using arterial spin-labeling MRI with multiple postlabeling delays: comparison with [15O]-gas PET and DSC-MRI. AJNR Am. J. Neuroradiol..

[7] Kuwabara Y (1997). Cerebral hemodynamics and metabolism in moyamoya disease–a positron emission tomography study. Clin. Neurol. Neurosurg..

[8] Kashiwagi S (1996). Regression of moyamoya vessels and hemodynamic changes after successful revascularization in childhood moyamoya disease. Acta Neurol. Scand. Suppl..

[9] Schubert GA (2014). Perfusion characteristics of moyamoya disease: an anatomically and clinically oriented analysis and comparison. Stroke.

[10] Takahashi S (2015). Hemodynamic stress distribution reflects ischemic clinical symptoms of patients with moyamoya disease. Clin. Neurol. Neurosurg..

[11] Lee S (2018). Monitoring cerebral perfusion changes after revascularization in patients with moyamoya disease by using arterial spin-labeling MR imaging. Radiology.

[12] Fan AP (2017). Long-delay arterial spin labeling provides more accurate cerebral blood flow measurements in moyamoya patients: a simultaneous positron emission tomography/MRI study. Stroke.

[13] Yun TJ (2013). Effect of delayed transit time on arterial spin labeling: correlation with dynamic susceptibility contrast perfusion magnetic resonance in moyamoya disease. Invest Rediol..

[14] Yun TJ (2009). Childhood moyamoya disease: quantitative evaluation of perfusion MR imaging–correlation with clinical outcome after revascularization surgery. Radiology.

[15] Lin YH (2019). Standardized MR perfusion scoring system for evaluation of sequential perfusion changes and surgical outcome of moyamoya disease. AJNR Am. J. Neuroradiol..

[16] Mouridsen K (2006). Bayesian estimation of cerebral perfusion using a physiological model of microvasculature. NeuroImage.

[17] Hara S (2019). Bayesian estimation of CBF measured by DSC-MRI in patients with moyamoya disease: comparison with [15O]-gas PET and singular value decomposition. AJNR Am. J. Neuroradiol..

[18] Albers GW (2018). Thrombectomy for stroke at 6 to 16 hours with selection by perfusion imaging. N. Engl. J. Med..

[19] Nogueira RG (2018). Thrombectomy 6 to 24 hours after stroke with a mismatch between deficit and infarct. N. Engl. J. Med..

[20] Olivot JM (2009). Optimal Tmax threshold for predicting penumbral tissue in acute stroke. Stroke.

[21] Kim DY (2017). Infarct pattern and collateral status in adult moyamoya disease: a multimodal magnetic resonance imaging study. Stroke.

[22] Fan AP (2017). Long-delay arterial spin labeling provides more accurate cerebral blood flow measurements in moyamoya patients: a simultaneous positron emission tomography/MRI study. Stroke.

[23] Meijs M, Christensen S, Lansberg MG, Albers GW, Calamante F (2016). Analysis of perfusion MRI in stroke: to deconvolve, or not to deconvolve. Magn. Reson. Med..

[24] Rashad S, Saqr KM, Fujimura M, Niizuma K, Tominaga T (2020). The hemodynamic complexities underlying transient ischemic attacks in early-stage Moyamoya disease: an exploratory CFD study. Sci. Rep..

[25] Mukawa M (2016). First autopsy analysis of a neovascularized arterial network induced by indirect bypass surgery for moyamoya disease: case report. J. Neurosurg..

[26] Huang, A., Lee, C.W. & Liu, H.M. *Curve fitting criteria to determine arterial input function for MR perfusion analysis*. Venice, Italy: Proceedings of the 16th IEEE International Symposium on Biomedical Imaging; 10.1109/ISBI.2019.8759307 (2019).

[27] Østergaard L (2005). Principles of cerebral perfusion imaging by bolus tracking. J. Magn. Reson. Imaging..

[28] Koo TK, Li MY (2016). A guideline of selecting and reporting intraclass correlation coefficients for reliability research. J. Chiropr. Med..

[29] Suzuki J, Kodama H (1983). Moyamoya disease–a review. Stroke.

[30] Cheong LH, Koh TS, Hou Z (2003). An automatic approach for estimating bolus arrival time in dynamic contrast MRI using piecewise continuous regression models. Phys. Med. Biol..

[31] Madsen MT (1992). A simplified formulation of the gamma variate function. Phys. Med. Biol..

[32] Wu O (2003). Tracer arrival timing-insensitive technique for estimating flow in MR perfusion-weighted imaging using singular value decomposition with a block-circulant deconvolution matrix. Magn. Reson. Med..

[33] Straka M, Albers GW, Bammer R (2010). Real-time diffusion-perfusion mismatch analysis in acute stroke. J. Magn. Reson. Imaging..

