# Supplementary material for: Time to peak and full width at half maximum in MR perfusion: valuable indicators for monitoring moyamoya patients after revascularization
Source: Sci Rep. 2021 Jan 12;11:479. doi: 10.1038/s41598-020-80036-3 (PMC7804964; doi:10.1038/s41598-020-80036-3)
Supplement: Supplementary file 1 — Supplementary Information. [file 41598_2020_80036_MOESM1_ESM.pdf]

Supplementary information

Time to peak and full width at half maximum in MR perfusion: Valuable indicators for monitoring moyamoya patients after revascularization

Adam Huang[1], Chung-Wei Lee[2], Hon-Man Liu[2,3]*

[1]Department of Biomedical Sciences and Engineering, National Central University, Taiwan

[2]Department of Medical Imaging, National Taiwan University Hospital, Taipei, Taiwan

[3]Department of Medical Imaging, Fu Jen Catholic University Hospital, Fu Jen Catholic University, New Taipei City, Taiwan

*Corresponding author E-mail address: inr.liu@gmail.com, hmliu@ntu.edu.tw

TTP and FWHM Estimation Error Analysis

An AIF Ca(t) was simulated using a gamma-variate function:

$$C_a(t) = \begin{cases} 0 & t \le t_0 \\ a(t-t_0)^b e^{-(t-t_0)/c} & t > t_0 \end{cases}$$

where a = 1, t_0 = 10, b = 3, and c = 1.5 to render an input function with a shape and size that would typically be obtained using a standard injection scheme[16]. The residue function *R* is:

$$R(t) = \int_t^{\infty} h(\tau;\alpha,\beta)d\tau$$

where the transport function *h* belongs to the family of gamma distributions:

$$h(t;\alpha,\beta) = \frac{1}{\beta^{\alpha}\Gamma(\alpha)} t^{\alpha-1} e^{-t/\beta}, \qquad \alpha,\beta > 0$$

We let α = λ and β = CBV/(λ·CBF) to ensure that the mean transit time is equal to CBV/CBF. We created testing data by:

1) randomly sampling λ between $\log_{10}(\lambda = 1)$ and $\log_{10}(\lambda = 100)$
2) randomly sampling the delay parameter τ of R between 0 to 5 s
3) assuming CBV = 4% and randomly sampling CBF values between 10 and 70 ml/100g/min.

Concentration time curves C(t) were generated by:

$$C(t_i) = CBF \cdot \Delta t \cdot \sum_{j=1}^{i} C_a(t_j)R(t_i - t_j)$$

with Δt = 0.1 s. Ground truth TTP and FWHM were then estimated with spline interpolated C(t) using temporal resolution of 0.01 s.

DSC-MRI signal curves S(t) were generated using $S(t) = S_0 \cdot \exp(-K \cdot C(t) \cdot TE)$ with S_0 =100 and TE = 65 ms. The constant *K* was chosen such that a 40% signal drop relative to the baseline S_0. All signal curves were down-sampled to Δt = 2 s and added with zero mean Gaussian noise to produce noisy data S_n with SNRs of 20, 26, 40:

$$S_n = S + A_n \cdot 40 \cdot \text{gaussian\_noise}(\text{mean} = 0, \text{std} = 1)$$

where the noise amplitude ratio A_n = 0.1, 0.05, and 0.01 respectively.

Figure S.1 illustrates the noisy signal examples S_n with SNRs of 20, 26, 40. Figure S.2 illustrates the correlations between estimated and ground truth TTP/FWHM with the Pearson correlation coefficients.

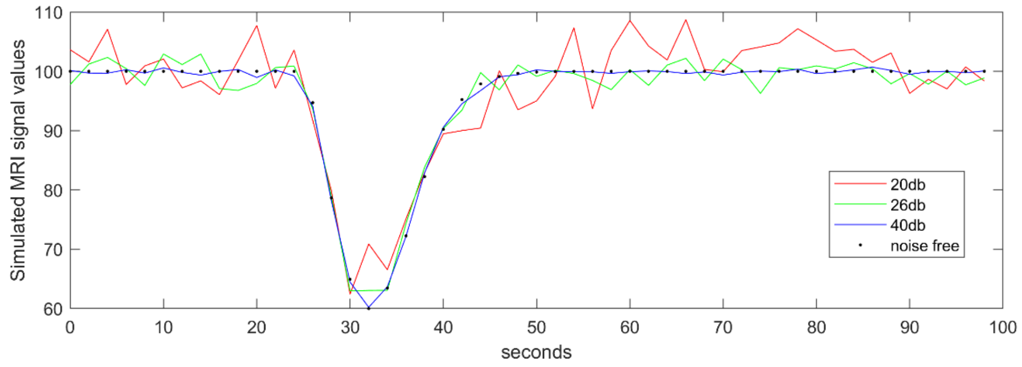

Figure S.1 Simulated noisy DSC-MRI signals with SNRs 20, 26, and 40 db.

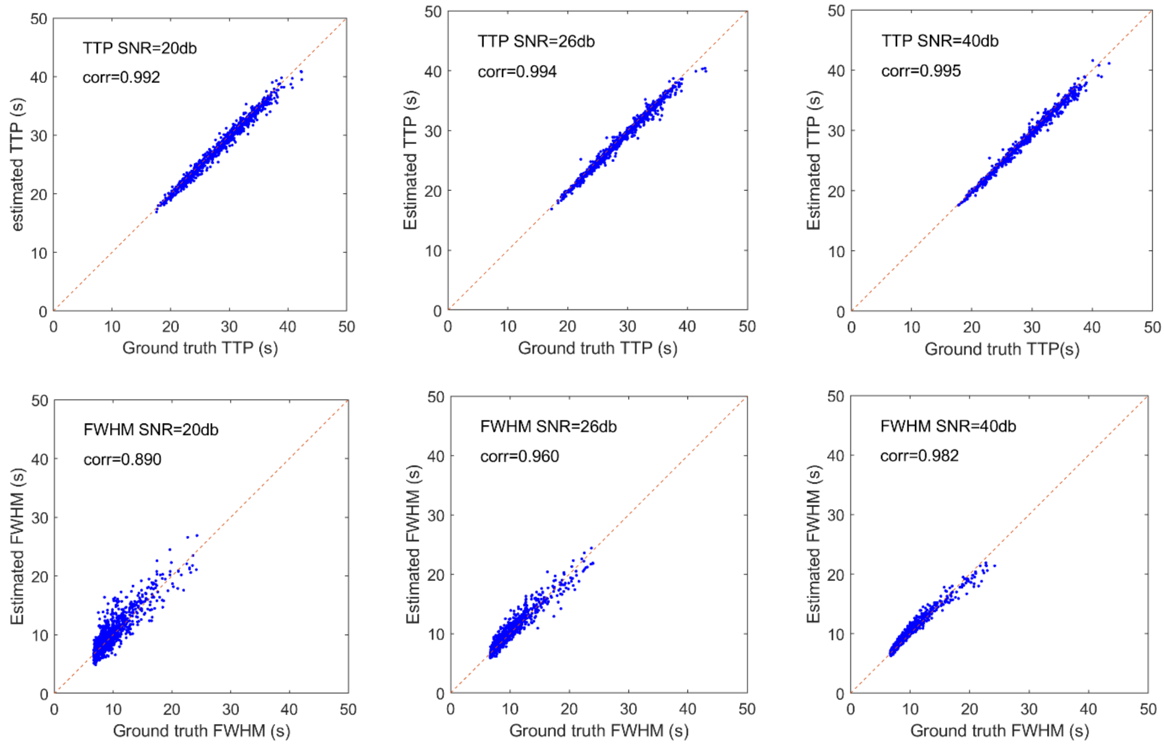

Figure S.2 GVM-based TTP and FWHM estimation error analysis from 1000 simulated samples with signal to noise ratio (SNR) levels at 20, 26, and 40 db. Their performances are evaluated by Pearson correlation coefficients (corr) respectively.

Case 13 perfusion maps

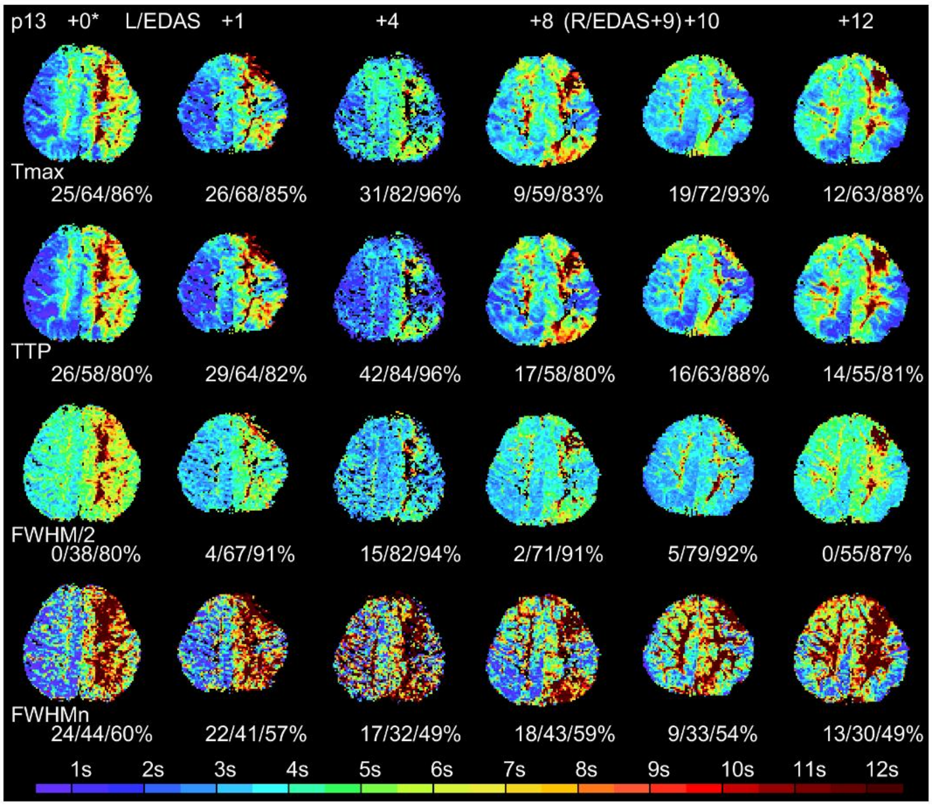

Figure S.3 Case 13 appears with no TTP and $FWHM_n$ improvement in Figure 2, which are evaluated based on the +0 and +12 scans only. Take a close look at the whole dataset, we observe that Tmax and TTP improve in the +4 and +10 scans. However, the surgical operation performed in the +9 month changes the +10 and +12 perfusion maps again. We need further scans to evaluate this case more accurately.

Case 18 perfusion maps

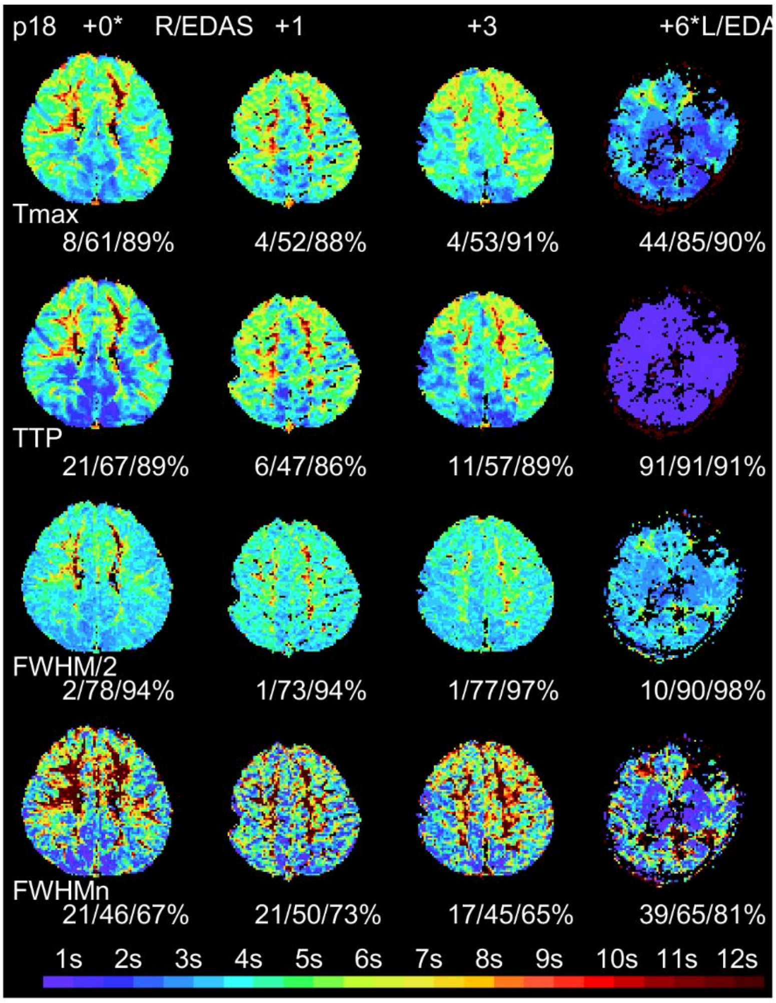

Figure S.4 Case 18 appears with no TTP and $FWHM_n$ improvement in Figure 2. Since the latest scan at the +6 month is very noisy, the evaluation done in Figure 2 is based on the +0 and +3 scans, which are relatively short (3 months apart) as compared to other cases.
